# Protective Role of Morin, a Flavonoid, against High Glucose Induced Oxidative Stress Mediated Apoptosis in Primary Rat Hepatocytes

**DOI:** 10.1371/journal.pone.0041663

**Published:** 2012-08-10

**Authors:** Radhika Kapoor, Poonam Kakkar

**Affiliations:** CSIR-Indian Institute of Toxicology Research, Lucknow, India; University of Pecs Medical School, Hungary

## Abstract

Apoptosis is an early event of liver damage in diabetes and oxidative stress has been linked to accelerate the apoptosis in hepatocytes. Therefore, the compounds that can scavenge ROS may confer regulatory effects on high-glucose induced apoptosis. In the present study, primary rat hepatocytes were exposed to high concentration (40 mM) of glucose. At this concentration decreased cell viability and enhanced ROS generation was observed. Depleted antioxidant status of hepatocytes under high glucose stress was also observed as evident from transcriptional level and activities of antioxidant enzymes. Further, mitochondrial depolarisation was accompanied by the loss of mitochondrial integrity and altered expression of Bax and Bcl-2. Increased translocation of apoptotic proteins like AIF (Apoptosis inducing factor) & Endo-G (endonuclease-G) from its resident place mitochondria to nucleus was also observed. Cyt-c residing in the inter-membrane space of mitochondria also translocated to cytoplasm. These apoptotic proteins initiated caspase activation, DNA fragmentation, chromatin condensation, increased apoptotic DNA content in glucose treated hepatocytes, suggesting mitochondria mediated apoptotic mode of cell death. Morin, a dietary flavonoid from *Psidium guajava* was effective in increasing the cell viability and decreasing the ROS level. It maintained mitochondrial integrity, inhibited release of apoptotic proteins from mitochondria, prevented DNA fragmentation, chromatin condensation and hypodiploid DNA upon exposure to high glucose. This study confirms the capacity of dietary flavonoid Morin in regulating apoptosis induced by high glucose via mitochondrial mediated pathway through intervention of oxidative stress.

## Introduction

Diabetes mellitus is a chronic metabolic disorder due to relative deficiency of insulin secretion, action or both. It affects nearly 230 million people worldwide and the number is expected to increase to around 346 million people by 2030 [1]. Diabetic condition induces state of hyperglycemia, characterized by high circulating blood glucose leading to functional and structural paucity [2]. Hyperglycemia leads to various complications such as neuropathy, nephropathy, retinopathy and many more. Although liver is the main organ of glucose homeostasis and holds importance during the progression of diabetes, not many studies have focussed on the development and progression of liver cell damage.

Literature suggest that hyperglycemia induced overproduction of reactive oxygen species (ROS) in mitochondria due to involvement of four important metabolic pathways including PKC (protein kinase C), hexosamine , advanced glycation and polyol pathway [3]. This condition can, in turn, induce various metabolic dysfunctions and contribute to progressive development of micro- and macro vascular complications and multi-organ damage. Hence, inhibition of ROS generation due to excess glucose can be an important strategy independently to restore the normal functioning of liver and control the progression of high glucose induced liver damage. Recent scientific reports indicate that oxidative stress is the biochemical trigger for hepatic dysfunction in diabetic rats [4], which in absence of strong defence by antioxidants can lead to activation of the stress –mediated intracellular signaling pathway.

One of the major cellular responses to high glucose induced stress and hence ROS generation in mitochondria is apoptotic cell death [5], [6] including apoptosis in liver. Most common and stereotypical death phenomena of a cell undergoing apoptosis includes shift and release of apoptotic proteins from their resident places, DNA fragmentation, nuclear condensation, morphological changes in cell and externalization of phosphatidylserine [7]. Caspases which are cysteine rich proteases are considered to play a crucial role during the execution of above stated events [8]. Mitochondria-dependent step, following outer membrane permeabilization, is associated with most pro-apoptotic stimuli. With loss of mitochondrial integrity the pro- and anti-apoptotic members of the Bcl-2 (b-cell Lymhoma-2) family gets involved and leads to the translocation of various apoptotic proteins that can trigger either caspase activated or caspase-independent death pathways [9], [10]. Certain caspase-independent death effectors like apoptosis inducing factor (AIF) and endonuclease G (Endo-G) have been reported to be localised in mitochondria [11]. The exact mechanism involved in hyperglycemia induced liver cell damage has still not been completely delineated.

Management of hyperglycemia by chemical drugs or insulin involves various drawbacks such as that induced by sulfonyl ureas and chronic anorexia nervosa, brain atrophy and fatty liver induced by insulin [12]–[13]. Hence an effective and economic way for better management of hyperglycemia can be by silencing or quenching the excess ROS generated by the use of various known natural antioxidants. These natural antioxidants are safe and easily acceptable by the cell systems.

Morin (3,5,7,2′,4′-pentahydroxyflavone (Figure S1), a natural bioflavonoid, and a major component of traditional medicinal herbs was originally isolated from members of the *Moraceae* family and is a constituent of many herbs, fruits and wine [14]. Previous researches have shown antioxidant, anti-inflammatory, and anti-proliferative effects of morin *in vivo* and *in vitro*. Morin is a member of the flavonoid family which is found in almond (*Prunus dulcis*), fig (*Chlorophora tinctoria*) and other Moraceae family plants extensively used as food and traditional herbal medicine [15]. Morin is also found in *Psidium guajava* (Indian guava). Guava is traditionally considered to be an effective antidiabetic plant with known antioxidant properties [16]. Since morin is the active component of guava, authors expect morin to exhibit same beneficial properties.

In our previous studies, we established the optimum glucotoxic dose for primary rat hepatocytes. So, the present study aims to investigate the mechanism- of cell death in primary rat hepatocytes due to the exposure of high concentration of glucose in which alterations in endogenous antioxidative systems and regulation of stress-sensitive signaling pathways are of prime importance. Capacity of morin to provide protection against hyperglycemia induced changes in apoptotic pathway in primary rat hepatocytes has been studied here.

## Materials and Methods

### Animals

Male Wistar rats weighing 180±20 g from Indian Institute of Toxicology Research (IITR) animal colony were used in the study. Rats were kept under standard conditions of 25±2°C temperature, 60–70% humidity and a controlled 12 h light/dark cycle. Rats were given standard pellet diet (Ashirwad Pellet Diet, Mumbai, India) and water *ad libitum*. Chloroform was used for euthanasia. Hepatocytes were isolated from overnight fasted rats. Animal handling in all experimental procedures was approved by the Institutional Animal Ethics Committee (IAEC) of Indian Institute of Toxicology Research (*formerly Industrial Toxicology Research Centre; ITRC*) with approval number (ITRC/IAEC/20/2010).

### Cell Culture

Rats were fasted overnight and euthanized. Primary hepatocytes were isolated according to the two step collagenase perfusion method [17]. Trypan blue dye exclusion method was used to access cell viability within 1 h of cell isolation. Only preparations with cell viability greater than 95% were used for subsequent experiments. Hepatocytes were maintained in RPMI-1640 media supplemented with heat inactivated 10% fetal bovine serum and 1% of 10,000 units Penicillin, 10 mg Streptomycin, 25 µg Amphotericin B, 1 mM sodium pyruvate, 2 mM glutamine under an atmosphere of 5% CO_2_-95% air in an incubator (Thermo-forma) with controlled humidity at 37°C. The cells were seeded at a density of 1.0×10^4^ cells/well (counted on hemocytometer) in 0.1% collagen pre-coated 96 well plates, and used for experiments after being cultured for 24 h. Morin hydrate (95% purity) was purchased from Sigma (St. Louis, MO, USA). Three different treatment regimens were used (Figure S2). In pre treatment, 5 µg morin was given to the cells for 30 min, change of media followed by 1.5 hr exposure to high glucose (40 mM). In co-treatment cells were exposed to morin and high glucose simultaneously for 1.5 hr. In post treatment after the high glucose exposure for 1.5 hr, media was changed and morin was supplemented for 30 min before cells were harvested. Illustration of treatment schedule is given below. Cells were cultured with high concentrations of glucose to mimic the hyperglycemic condition [11], [12]. The cytotoxic dose of glucose i.e.40 mM glucose has been apparently used to depict hyperglycemic condition by many researchers [18]–[23], in addition to it, 40 mM glucose concentration has been reported in neonates and adult diabetic patients. Leinninger et al have reported that high glucose can induce apoptosis within 30 min exposure time [19]. To exclude a hyper-osmolar effect, identical concentration of mannitol was added in control cells.

### Cell viability assay by Alamar blue

The active ingredient of Alamar blue (resazurin) is a non-toxic, cell permeable compound that is blue in color and virtually non-fluorescent. Upon entering cells, resazurin is reduced to resorufin, which is a highly fluorescent red compound. Viable cells continuously convert resazurin to resorufin, thereby generating a quantitative measure of viability. Alamar blue assay was done according to manufacturer's protocol (Invitrogen, Carlsbad, CA). Fluorescence intensity was measured on a spectrofluorometer (Varioskan Flash, Thermo) at excitation and emission wavelengths of 530 nm and 570 nm, respectively.

### LDH activity based cytotoxicity assay

LDH release into the media was taken as an indicator of cell damage and was measured with an assay kit Tox-7 Sigma (Saint Louis, MO, USA). The assay is based on the principle of reduction of NAD by LDH. The reduced NAD (NADH) is utilized in the stoichiometric conversion of a tetrazolium dye which is measured spectrophotometrically. After treatment was over, the 96-well plate was centrifuged at 240 g for 4 minutes and culture supernatant was transferred in a new plate. The assay mixture was prepared and added to each well and the plate incubated wrapped in foil at room temperature for 30 min. Reaction was terminated by adding the stop solution to each well. The plate was read at 490 nm at a reference wavelength of 690 nm. The extent of LDH leakage is expressed as the fold of absorbance of control [24].

### Antioxidant status

#### Superoxide dismutase activity

SOD (superoxide dismutase) activity was done according to Kakkar *et al.*, [25]. SOD assay is based on the spectrophotometric assessment of the inhibition of nitro blue tetrazolium-NADH and phenazine methosulphate (PMS) mediated formazan formation. Absorbance was measured at 560 nm. 50% inhibition of formazan formation under the assay condition in 1 min is taken as one unit of enzyme activity/minute.

#### Catalase activity

CAT (catalase) was assayed spectrophotometrically using the method of Aebi *et al.*, [26]. Assay is based on the principle of measurement of decomposition of H_2_O_2_ by catalase measured at 240 nm. Catalase activity is expressed as µmole H_2_O_2_ decomposed/min/10^4^ cells.

#### Reduced Glutathione (GSH)

GSH is considered as the natural antioxidant of the cell. GSH is a major intracellular antioxidant performing several important biological functions. The method described by Dringen and Hamprechit *et al.*, [27] with certain modifications was used to measure the total GSH spectrophotometrically in a cellular system. 50 µl of cell lysate was diluted with 50 µl of 100 mM phosphate buffer containing 1 mm EDTA (ethylenediamine tetraacetic acid). To this mixture 100 µl of reaction buffer 295 µM 5, 5′-dithio-bis (2-nitrobenzoic acid) (DTNB) made in 10 ml of phosphate buffer], was added and measured at 412 nm within 5 min. GSH from Sigma was employed to obtain a standard curve. Reduced GSH is expressed as µM GSH/10^4^ cells.

#### Glutathione Peroxidase

The method of Paglia and Valentine [28] was used to measure glutathione peroxidase activity. Assay mixture contained 2.525 ml of 0.1 mol/L tris HCl buffer (pH 7.2), 75 µl of 0.04 mol/L GSH, 100 µl of 0.1 mol/L nicotinamide adenine dinucleotide phosphate (NADPH) and 100 µl of Glutathione reductase (0.24 units). 15–20 µl of cell lysate was added to the reaction mixture. Reaction was initiated by adding 100 µl of 0.75 mmol/L hydrogen peroxide. The decrease in absorbance was measured at 340 nm for 3 minutes at every 30 sec. The activity was expressed as nmol NADPH oxidized/mg protein/min using molar extinction coefficient of 6.22×10^3^(mmol/L)^−1^ cm^−1^.

### Measurement of ROS production in the cells

Fluorescent probe DCFH-DA (2′, 7-dichlorofluoresceindiacetate) was used to measure ROS generation in hepatocytes by the method of Zhang *et al.*, 2008 [29]. Upon entering the cell, the diacetate bond of the fluoroprobe is cleaved by intracellular esterases leaving DCFH which is oxidized to DCF (dichlorofluorescein) by the oxidants and its fluorescence is taken as an indicator of ROS production in the cell. The fluorescence intensity was measured on a spectrofluorometer (Varioskan Flash, Thermo) at excitation and emission wavelengths of 485 nm and 530 nm, respectively.

### Expression of antioxidant, apoptotic and antiapoptotic genes

Total RNA was isolated from treated and un-treated hepatocytes by using the Trizol reagent (Invitrogen, Carlsbad,CA, USA) according to the manufacturer's instructions. 3 µg of total RNA was reverse transcribed (RT) into cDNA using Revert Aid H minus First Strand cDNA Synthesis Kit (Fermentas, EU) as per manufacturer's instructions. After reverse transcription, cDNA was used for semi-quantitative PCR using sets of specific primers as shown in Table 1. cDNA amplification was carried out according to the respective temperature profile and number of cycles for Bax (bcl-2 associated X), Bcl-2, Caspase3, Caspase 9, GPx (glutathione peroxidase), GR, Mn-SOD and Cu Zn-SOD. GAPDH (glyceraldehydes-3 phosphate dehydrogenase) was used as internal control. 5 µL of the product was run on 1.5% agarose gel, and photographed on UV transilluminator (Alfa-Innotech; CellBio- 264 sciences Inc., SantaClara,CA) using a digital camera. PCR products were analyzed using Image J software 1.44 (USA) and normalized to GAPDH.

**Table 1:** Primers used for expression of apoptotic, anti-apoptotic and antioxidant genes and their expected product size.

Genes	Primers	Product size
**Bcl-2**	F 5′-ACTTTGCAGAGATGTCCAGTCAG-3′R 5′-GTTCAGGTACTCAGTCATCCACAG-3′	458
**Bax**	F 5′-GGAGGAAGTCCAGTGTCCAG-3′R 5′-TGCAGAGGATGATTGCTGAC-3′	172
**GPx**	F 5′-CAGTTCGGACATCAGGAGAAT-3′R 5′-AGAGCGGGTGAGCCTTCT-3′	290
**GR**	F 5′-GGGCAAAGAAAGATTCCAGGTT-3′R 5′-GGAGGCTTCATCTTCAGTGA-3′	252
**MnSOD**	F 5′-CGTGCTCCCACACATCAATC-3′R 5′-TGAACGTCACCGAGGAGAGA-3′	240
**CuSOD**	F 5′-CGTCATTCACTTCGAGCAGA-3′R 5′-CACCTTTGCCCAAGTCATCT-3′	438
**Caspase-3**	F 5′- GAACGAACGGACCTGTGGACCT-3′R 5′-GCCTCCACTGGTATCTTCTGGCAT-3′	187
**Caspase-9**	F 5′-TGAGCCAGATGCTGTCCCATACCAG-3′R 5′-CCTGGGAAGGTGGAGTAGGACAC-3′	114
**GAPDH**	F 5′-GGCCAAGATCATCCATGACAACT-3′R 5′-ACCAGGACTGAGCTTGACAAAGT-3′	462

### Mitochondrial Membrane Potential

Flouroprobe JC-1 (5,5′,6,6′-tetrachloro-1,1′,3,3′-tetraethylbenzimidazol-carbocyanine iodide) has been extensively used to study the loss of the mitochondrial membrane potential which occurs during apoptosis. JC-1 exists in its monomeric form, giving green fluorescence in cells with low membrane potential (depolarized mitochondria). In cells with high membrane potential (polarized mitochondria), formation of dimers of the dye is promoted, resulting in red fluorescence. A decrease in red/green ratio is indicative of apoptosis. Cultured cells were incubated with 2.5 µg/ml of JC-1 flouroprobe at 37°C in dark for 30 min. After washing the hepatocytes, ΔΨm was assessed by comparing two fluorescence, i.e. red (Ex/Em-580/590 nm)/green (Ex/Em-510/527 nm) using Varioskan fluorescent microplate reader [30].

### Preparation of Sub-cellular fractions

From control and treated primary rat hepatocytes, nuclear fraction was obtained as described by Leal *et al.*, 2009 [9] whereas cytosolic and mitochondrial fractions were prepared as described by Tripathi *et al.*, 2010 [24].

### Immunoblot analysis

The protein content corresponding to each treatment was quantified using Lowry's method [31]. Sixty microgram of protein sample from nuclear, cytosolic or mitochondrial fraction was separated by electrophoresis on 12% SDS– polyacrylamide gel and electro blotted on PVDF (polyvinylidene difluoride) membrane (HybondTM –P Amersham Biosciences, UK limited, NA). After blocking non-specific sites with 1× blocking buffer, washing was performed using TBS (Tris buffer saline) containing 0.1% Tween-20. The membrane was then incubated for 1 h with specific polyclonal IgG antibodies of Cyt c (cytochrome c), AIF, Endo-G, Cox IV, Lamin and β-Actin (Santa Cruz Biotechnology, Inc.) in 1∶500 dilutions. This was followed by washing with PBS (phosphate buffer saline) and incubation with horse-radish peroxidase-conjugated Rabbit anti-goat IgG, anti-rabbit IgG or goat anti-mouse IgG secondary antibodies in 1∶1000 dilutions for 1 h at room temperature. Membrane was rewashed and the immunoblot was revealed using Immobilon Western Chemiluminescent HRP (horseradish peroxidase) substrate kit. (Millipore, Corporation, MA, USA). PageRuler Prestained Protein Ladder (5 µl), (SM-0671 from Fermentas, EU) was used to determine molecular weight of the protein bands. Densitometry of the bands obtained was done using NIH software Image J version 1.41 (USA). Band areas were calculated by densitometric scanning and result expressed as Arbitrary Units for each experimental band.

### Caspase-3 Enzymatic activity

Method described by Rodrigues et al was used to estimate caspase-3 enzymatic activity. Enzymatic cleavage of chromophore *p*-nitroanilide (pNA) from the substrate *N*-acetyl- Asp-Glu-Val-Asp-pNA (Sigma) denotes the general caspase activity. The reaction mixtures were incubated at 37°C for 1 h, and the formation of pNA was measured at 405 nm with a 96-well plate reader [30].

### Determination of apoptosis

#### Apoptotic DNA Content

To determine the population of apoptotic cells, analysis was done using propidium iodide (PI) staining. Hepatocytes were fixed using 70% ethanol and further permeabilized with 0.1% Triton X-100. Cells were again washed with PBS and resuspended in PBS containing 50 mg/ml PI and 1 mg/ml RNase A for 30 min in the dark at 4°C. Labeled nuclei were then analysed on flow cytometer and then gated to remove debris. The percentage of nuclei with sub-G1 content was considered apoptotic cells. PI fluorescence was measured through a FL-2 filter (585 nm) [32].

#### DNA fragmentation

DNA was isolated from control and treated hepatocytes as described by Tripathi *et al* [24]. DNA quantification was done spectrophotometrically at 260/280 nm on NanoDrop spectrophotometer Q20 (ND-1000; NanoDrop Technologies, Inc., USA). DNA samples were finally separated by electrophoresis on 1.8% agarose gel with Tris–Borate/EDTA buffer and analyzed on Alfa-Innotech; CellBio- 264 sciences, Inc. SantaClara,CA image analyzer.

#### Fluorographic monitoring of apoptosis associated nuclear alterations

The nuclear condensation was observed using bisbenzimide (Hoechst 33258) fluorochrome. Primary hepatocyte monolayer (treated and control) were fixed in 4% paraformaldehyde for 10 min. Cells were then stained with Hoechst 33258 (5 µg/ml) for 10 min followed by washing and mounting in a solution of 90% glycerol (pH 5.5). Apoptosis associated nuclear alterations were examined at a wavelength of Ex/Em-350/460 nm using Nikon microscope (TS 80i, Leica, Wetzlar, Germany) with fluorescence attachment [24].

### Statistical Analysis

Data are expressed as mean ± SE. Data was analyzed on SPSS software version 14.0 using one-way ANOVA followed by student's t-test. *P<0.05, **P<0.01, ***P<0.001 were used as the criterion for significance.

## Results

### Morin protects against high glucose induced cytotoxicity and LDH release

**Figure 1:**
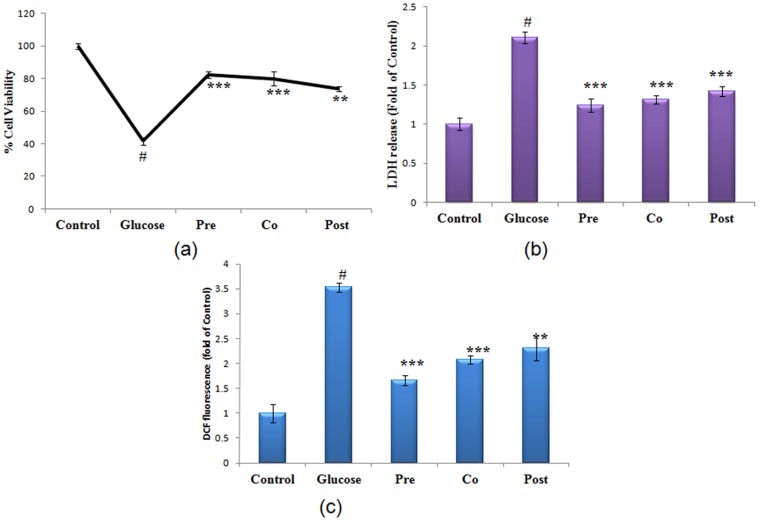
Protection accorded by morin during high glucose cytotoxicity. (a) Effect of morin on cell viability of glucose stressed primary hepatocytes as assessed by Alamar Blue. Relative viability is shown as the percentage of viable cells compared to control. (b) LDH release was assessed in control, high glucose treated (40 mM) and morin treated (pre, co and post-treated) stressed cells, expressed as the fold change compared to untreated cells. (c) ROS generation assessed by spectrofluorometry using fluoroprobe DCFH-DA. Results are represented as fold of DCF fluorescence as compared to control during increasing concentration of glucose. Results are shown as mean ± S.E. # denotes significant difference compared with control values. *P<0.05, **P<0.01 and ***P<0.001 denotes significant difference compared with 40 mM glucose treated cells. Results are representative of three separate experiments. The S.D. was below ±5% in all cases.

Cell viability of hepatocytes treated with 40 mM of glucose and morin (5 µg) under different treatment regime (Pre/co/post-treatment) was studied. It was found that cell viability of glucose stressed hepatocytes decreased to 49.50% (P<0.001). Treatment with morin enhanced cell viability by 1.98 fold (P<0.001), 1.92 fold (P<0.001), 1.72 fold (P<0.01) in pre-exposure, co-exposure and post-exposure respectively, when compared to glucose treated cells (Fig. 1a). The cell membrane integrity was assessed by estimation of LDH in culture supernatant. There was increased LDH release by 2.2 fold (P<0.001) in high glucose treated cells. In the presence of morin there was a significant decrease in LDH release from cells, maximum reduction being 1.74 fold during pre-treatment (P<0.001) (Fig. 1b).

### Prevention of ROS generation by morin

Significant increase in intracellular ROS generation (2.5 fold) was observed when primary hepatocytes were exposed to high glucose stress as assessed by fluorescent dye DCFH-DA. Morin treated cells, under different exposure conditions, exhibited reduced ROS generation. Cells pre-treated with morin showed a highly significant (P<0.001) decline in the ROS generation by 2.33 fold in comparison to glucose stressed cells, whereas, 1.75 fold (P<0.001) and 1.6 fold (P<0.01) decrease in ROS generation was observed in co- treated and post- treated hepatocytes (Fig. 1c).

### Expression of antioxidant genes and modulation of antioxidants enzymatic activities

**Figure 2:**
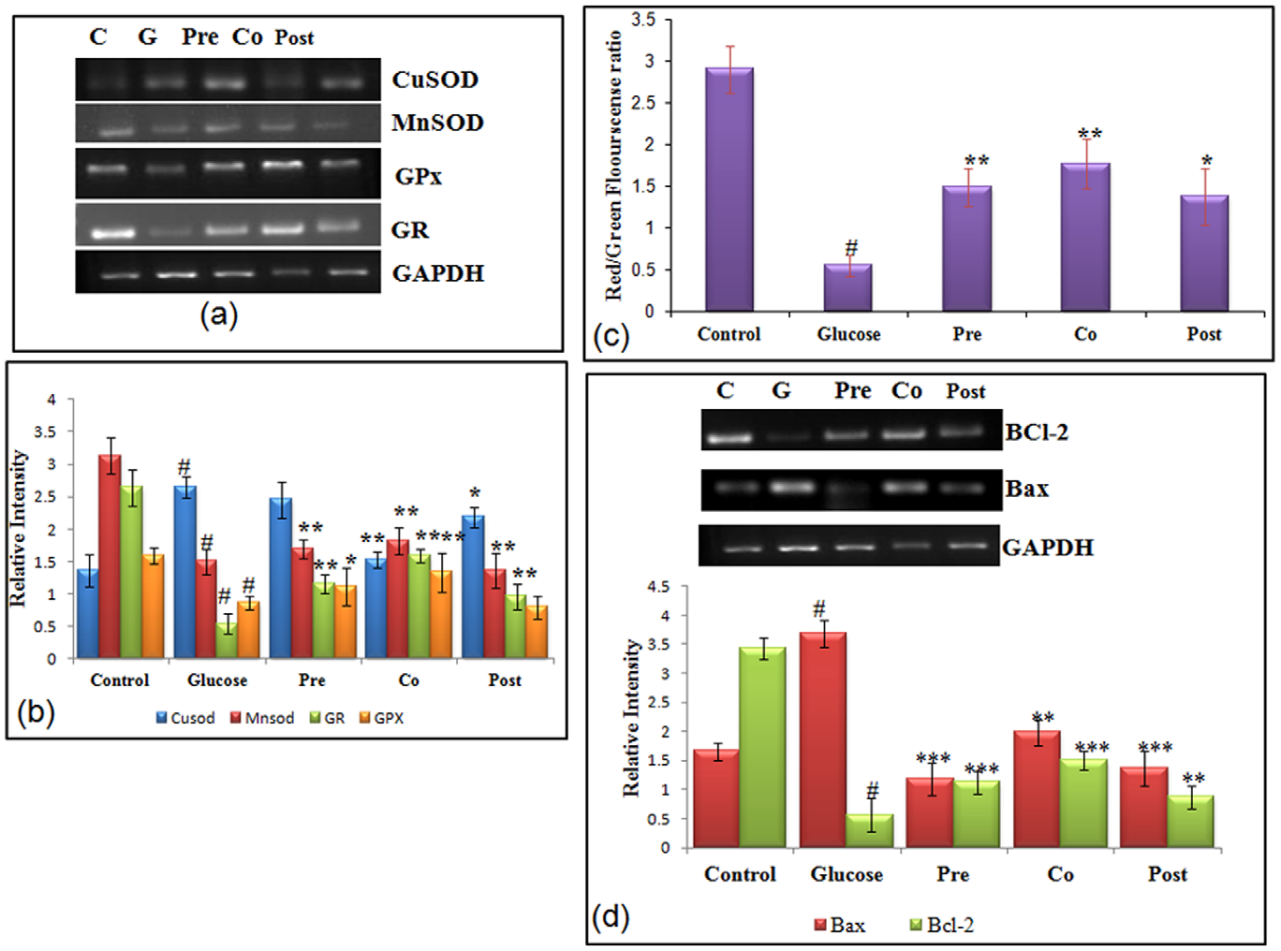
Effect of morin on antioxidant status, MMP and transcriptional level of Bax and Bcl-2. (a) Transcriptional level of antioxidant genes. m-RNA of MnSOD, Cu Zn-SOD, GPx and GR were amplified by RT-PCR and analyzed by electrophoresis on 1.8% agarose gel and ethidium bromide staining. GAPDH was used as internal control. (b) Bar graphs show fold change in expression of MnSOD, Cu Zn-SOD, GPx and GR. (c) Induction of mitochondrial membrane collapse in hepatocytes cultured with high glucose. MMP was assessed in control, high glucose treated (40 mM) and morin treated (pre, co and post-treated) stressed cells. Mitochondrial membrane potential of treated and control cells was assessed by fluorescent spectrophotometer using JC-1. Decrease in the red (polarized)/green (depolarized) fluorescence ratio reflects increased number of depolarized mitochondria. (d) m-RNA level of Bcl-2 and Bax, were amplified by RT-PCR and analyzed by 1.8% agarose gel electrophoresis followed by ethidium bromide staining. GAPDH was used as internal control. Graph shows fold change in m-RNA level. Results are shown as mean ± S.E. # denotes significant difference compared with control values and *P<0.05, **P<0.01 and ***P<0.001 denotes significant difference compared with 40 mM glucose.

To study the involvement of oxidative stress generated during high glucose stress, transcriptional level of antioxidant genes like MnSOD (manganese superoxide dismutase), CuZn-SOD (Copper zinc superoxide dismutase), GPx, and GR (glutathione reductase) was studied by RT-PCR (reverse transcriptase polymerase chain reaction), in addition to it and enzymatic activity of antioxidant enzymes was also studied. MnSOD is known to reduce mitochondrial ROS generation and Cyt-c release and hence is also considered an anti-apoptotic gene [33]. Hepatocytes cultured with high glucose showed decreased (1.83 fold, P<0.01) level of MnSOD. The effect of hyperglycemia was abrogated by administration of morin. An increase of 1.28 fold (P<0.01) was observed in the level of MnSOD in cells pre-treated with morin. Level of Cu Zn-SOD was found to enhance by 2.14 fold in 40 mM glucose treated hepatocytes, which further decreased by 1.96 fold in cells co- treated with morin. In stressed cells, Cu Zn-SOD possibly increased as a defence mechanism to overcome the persistent oxidative stress. Expression of other antioxidant genes like GPx and GR was significantly reduced by 3.80 and 2.01 fold respectively on treatment with high glucose. Morin on account of its antioxidant capacity was able to decrease ROS generation and restored the level of antioxidant genes- GPx and GR to values comparable to control. m-RNA level of GPx and GR which decreased as an effect of high glucose was subsequently increased by 2.43 fold and 1.80 fold respectively in cells co-treated with morin (Fig. 2a & 2b). Corresponding to the results obtained in transcriptional studies, high glucose significantly altered the GSH level and antioxidant enzymatic activity of hepatocytes (Table 2). High glucose significantly (P<0.001) decreased GSH level (3.16 fold); SOD activity (2.26 fold); catalase activity (2.1 fold) and GPx activity (2.5 fold). GSH level and enzymatic activity of antioxidant enzymes were significantly restored in cells treated with morin. Response seen in pre- and co- treated cells was found to be similar and significant restoration of the antioxidant level (P<0.001) was observed.

**Table 2:** Modulation of antioxidant status by morin in primary rat hepatocytes subjected to glucose stress.

Treatment	SOD activity (units/min/10^4^cells)	Catalase activity (mM H_2_O_2_ decomposed/min/10^4^ cells)	GPx activity (µM NADPH decomposed/min/10^4^cells)	GSH level (µMGSH/10^4^cells)
**Control**	1.26±0.10	13.01±0.70	20.86±1.05	9.57±0.29
**Glucose**	0.61±0.10#	5.02±0.54#	9.01±0.61#	2.86±0.48#
**Pre**	1.10±0.06***	9.04±0.92***	17.88±1.65***	7.91±0.41***
**Co**	1.19±0.05***	9.47±0.38***	17.53±2.37***	8.14±0.40***
**Post**	0.92±0.09***	6.60±0.35**	12.85±1.36**	5.66±0.47**

Results are shown as means ± S.E. from three independent experiments.

# denotes significant difference compared with control value.

*P<0.05,

** P<0.01 and

*** P<0.001 denotes significant difference from glucose, n = 6.

### Effect of morin on Mitochondrial Membrane Potential (ΨΔm) in cells under glucose stress

Increased ROS generation within the cell and depleted antioxidant defence marks the initiation of mitochondrial dysfunction and depolarisation. Mitochondrial depolarization is the indication of improper functioning of mitochondria. Ability of glucose to alter the mitochondrial trans-membrane electrical potential was investigated in primary hepatocytes. As mitochondria get depolarized, the fluorescence of the JC-1 dye changes from red to green. An increase of monomeric JC-1 molecules (green fluorescence) due to a decrease of mitochondrial membrane potential occurred in glucose stressed hepatocytes. A 4.65 fold (P<0.001) decrease in the ratio of red to green fluorescence was observed in glucose stressed hepatocytes as the mitochondria became progressively depolarized. Treatment of cells with morin resulted in significant enhancement in red/green fluorescence with maximum increase of 3.48 fold observed in morin co-treated cells (Fig. 2c) indicating prevention of depolarization of mitochondria.

### Expression of Bax and Bcl-2

Following mitochondrial depolarization, Bcl2 family plays a crucial role in cell apoptosis. To further study the effect of high glucose on expression of the Bcl2 family genes, RT-PCR was carried out to assess the Bax and Bcl2 levels. Bax, a pro-apoptotic Bcl2 family member was found to increase significantly (P<0.001; 2.92 fold) and Bcl-2, anti-apoptotic gene was decreased significantly (P<0.001). Morin modulated the transcription of pro- and anti-apoptotic gene and significantly modulated the expression by 2.92 fold and 1.66 fold respectively (Fig. 2d).

### Translocation of Proteins

**Figure 3:**
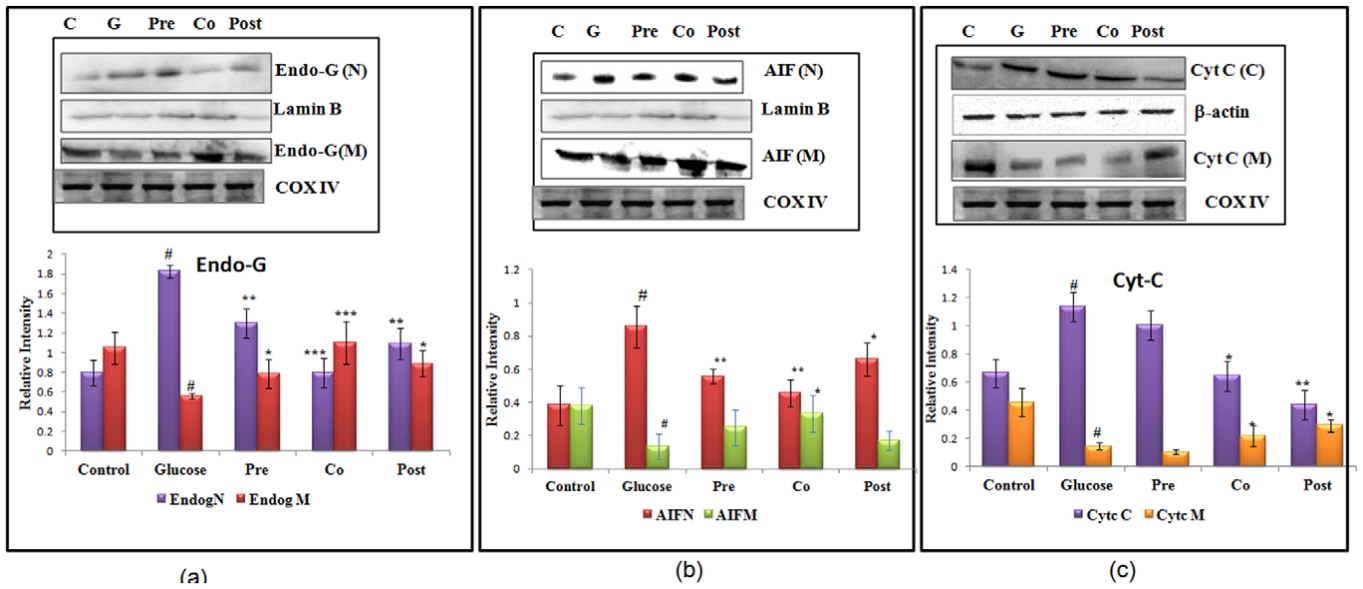
Translocation of apoptotic mitochondrial resident proteins. (a) Endo-G release from mitochondria and its translocation to nucleus. Cox (IV) served as internal control for mitochondria and Lamin-B served as internal control for nuclear fraction. (b) Changes in the immune-reactivity of AIF in the mitochondrial and nuclear fractions with COX IV and Lamin-B serving as control for mitochondrial and nuclear fractions respectively. (c) The changes in immunoreactivity for Cyt-c in the mitochondrial and cytosolic fractions. Mitochondrial Cyt-c was depleted significantly after high glucose treatment, with Cox (IV) serving as internal control. Cytosolic Cyt-c increased concomitantly upon high glucose treatment with *β*-actin serving as internal control. Results are shown as mean ± S.E. # denotes significant difference compared with control values and *P<0.05, **P<0.01, ***P<0.001 denotes significant difference compared with 40 mM glucose. Results are representative of three separate experiments.

Release of mitochondrial proteins like Cytochrome c, Endo G and AIF (Apoptosis inducing factor) marks the major event during apoptosis. During apoptosis, AIF and Endo-G (endonuclease-G) are translocated from mitochondria to nucleus, where they are involved in nuclear condensation and DNA damage. Mitochondria are reported to contain death effectors like Endonuclease G which during apoptosis translocates to nucleus, where it causes oligo-nucleosomal DNA fragmentation. Hepatocytes when incubated with high concentration of glucose showed 2.42 fold increase in the levels of Endo-G in nuclear fraction (Fig. 3a). Significant reduction of 3.85 (P<0.001) fold was observed in level of nuclear Endo-G with co-treatment of morin. Hepatocytes incubated with high glucose showed 3.20 fold decrease in the level of AIF in mitochondria and a substantial increase (3.10 fold) in nuclear fraction. On treatment with morin, during pre- and co- treatment schedules, the level of AIF was found to be enhanced in mitochondrial fraction and decreased in nuclear fraction indicating prevention of its translocation (Fig. 3b). Translocation was restored by 4.01 fold on co-treating cells with morin. To analyze the translocation of Cyt-C, its protein level was quantified by western blot in mitochondrial and cytosolic fractions of control and treated hepatocytes. The level of Cytochrome c was depleted from its resident location i.e. mitochondria, after treatment of primary hepatocytes with high concentration of glucose and increased in cytosolic fraction. The altered level of Cytochrome c was restored on treatment with morin (Fig. 3c).

### High glucose induced change in caspase3/9 gene expression and caspase-3 enzymatic activity

**Figure 4:**
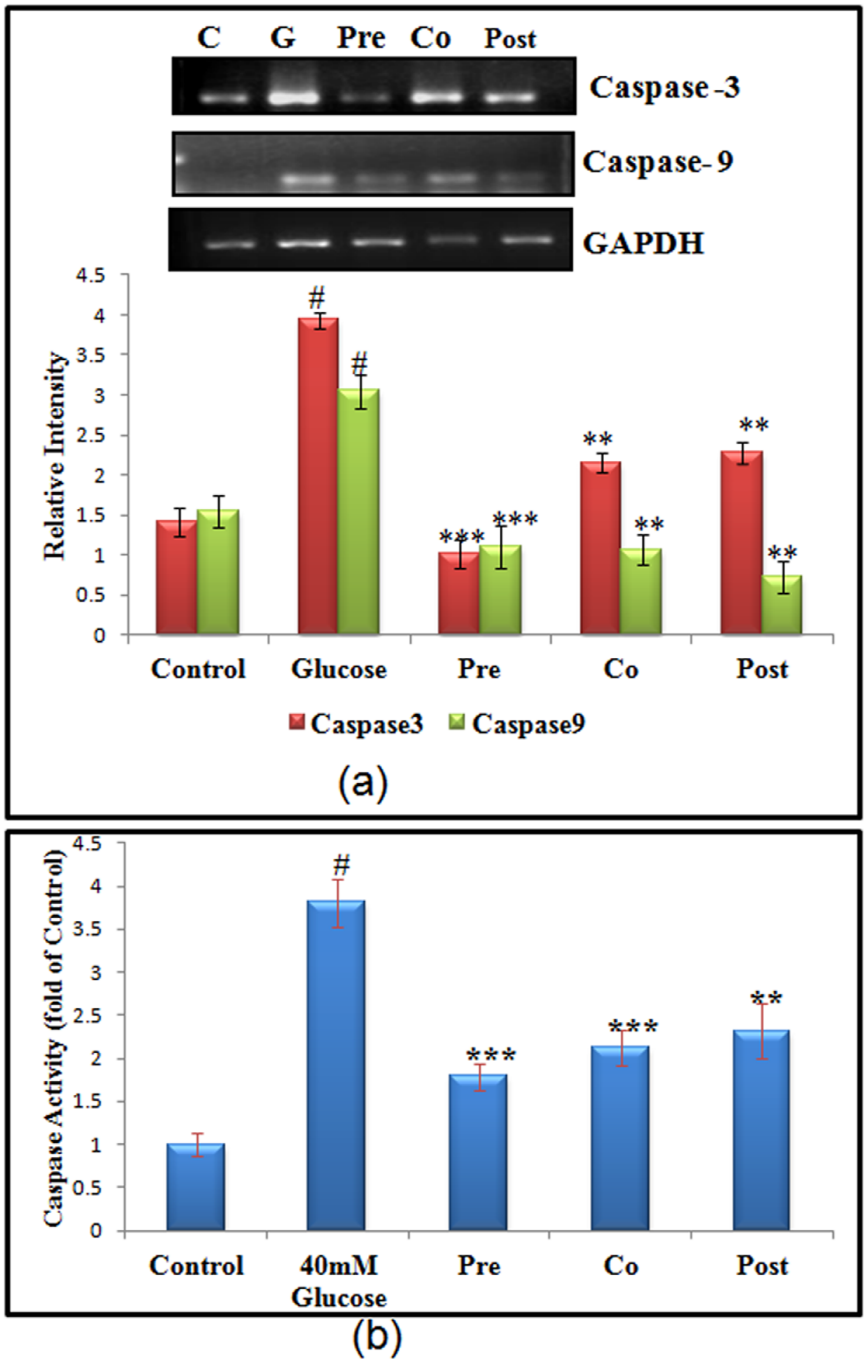
Effect of morin on m-RNA level of apoptotic genes. (a) m-RNA level of apoptotic genes such as caspase-3 and caspase-9 were amplified by RT-PCR and analyzed by 1.8% agarose gel electrophoresis followed by ethidium bromide staining. GAPDH was used as internal control. Graph shows fold change in m-RNA level. (b) Caspase -3 enzymatic activity of control and stressed cells. Results are shown as mean ± S.E. # denotes significant difference compared with control values and *P<0.05, **P<0.01 and ***P<0.001 denotes significant difference compared with 40 mM glucose.

Caspases play a central role in both induction as well as execution of apoptosis. Caspase- 9 and caspase-3 are the main effector and executor caspases involved in intrinsic pathway of apoptosis and play a critical role in the disintegration of the cells undergoing apoptosis. Caspase-9 gene expression was found to increase by 2.01 (P<0.001) fold during high glucose stress. Cells that were pre-treated with morin showed decrease in the caspase-9 expression by 3.32 fold (P<0.001). RT-PCR analysis of caspase-9/-3 activation was performed in primary cultured hepatocytes stressed with high glucose and treated with morin. Transcriptional level of caspase-3 revealed significant increase of 2.92 (P<0.001) fold in high glucose treated hepatocytes, which was decreased by 3.23 fold (P<0.001) on pre-treating high glucose stressed cells with morin (Fig. 4a). Results obtained in caspase-3 RT-PCR analysis corresponded to the results obtained by caspase-3 enzymatic activity. Hepatocytes exposed to high glucose showed increase of 3.23 fold in caspase-3 enzymatic activity, which was prevented in pre-treated cells with morin. Cells pre-treated with morin showed decreased caspase-3 activity by 2.02 fold (Fig. 4b).

### Detection of Apoptosis

#### DNA fragmentation

**Figure 5:**
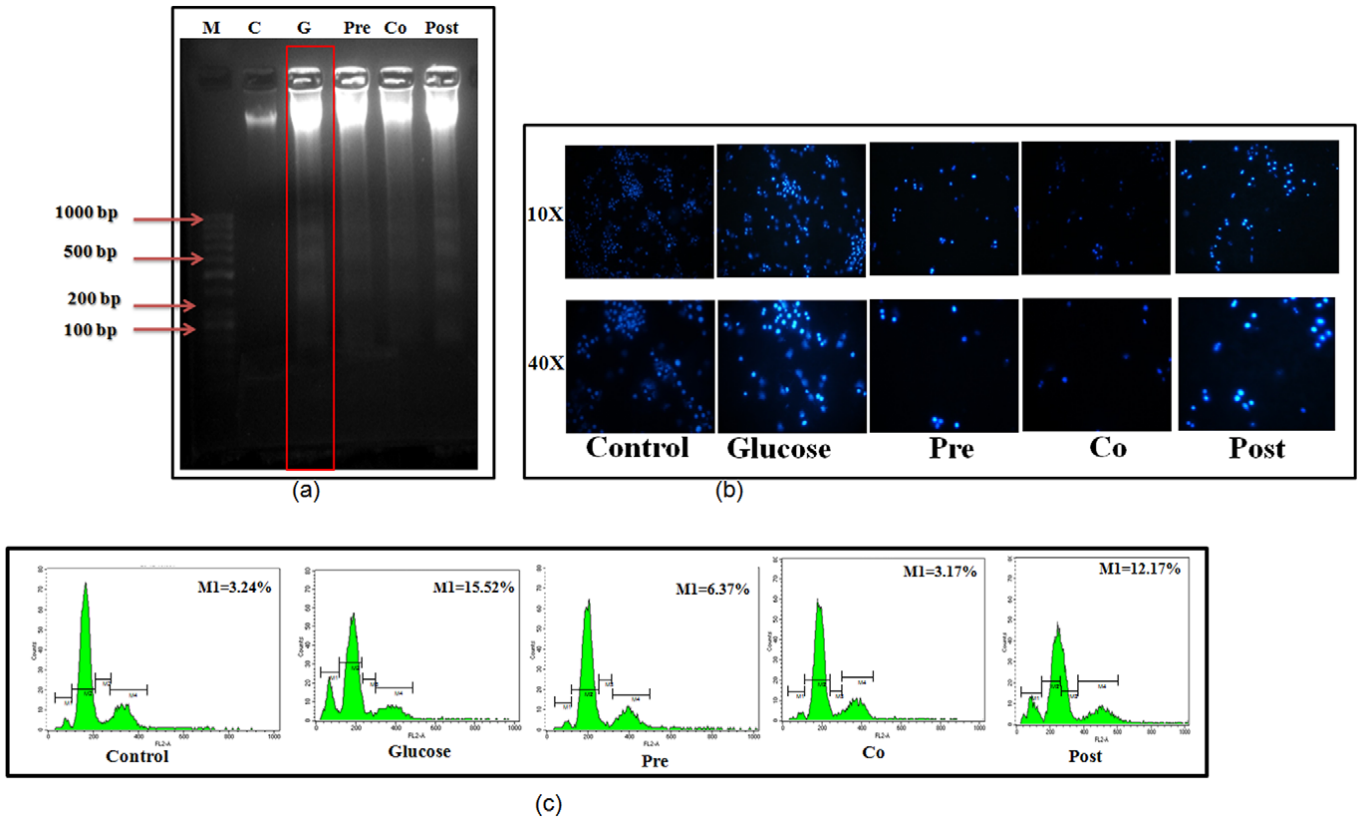
Modulation of hyperglycemia induced DNA damage, nuclear condensation and apoptosis by morin. (a) Intra-nucleosomal DNA fragmentation in control, high glucose treated (40 mM) and morin treated (pre, co and post-treated) stressed cells. Genomic DNA was isolated and analyzed on 1.8% agarose gel. Lane1 DNA Marker 100 bp: Lane 2: Control DNA, Lane 3: DNA from 40 mM Glucose treated hepatocytes, Lane 4: Morin pre-treatment, Lane 5: Morin Co-treatment, Lane 6: Morin Post treatment. Results are representative of three separate experiments. (b) Rat hepatocytes were stained with Hoechst 33258 and visualized at 10× and 40× magnifications to observe chromatin condensation. Results are representative of three separate experiments. (c) Effect of morin on hyperglycemia induced changes in cell cycle. Control, high glucose treated (40 mM) and morin treated (pre, co and post-treated) stressed cells were stained with propidium iodide and kept in dark for 30 minutes. Results expressed as the % of sub-G1 population. The propidium iodide fluorescence was measured using flow cytometer (Beckton Dickinson-LSR) with FL-2 filter. S.D. was below ±5% in all the cases. Results are representative of three separate experiments.

Translocation of AIF and Endo-G to nucleus causes nuclear condensation and DNA damage. To examine apoptosis in the hepatocytes under high glucose condition, inter-nucleosomal DNA fragmentation was assessed using electrophoresis. DNA samples from the control hepatocytes and hepatocytes pre and co treated with morin showed no distinct low molecular weight bands, but high molecular weight mass was retained on the top portion of the gel (Fig. 5a, lane 2, 4 and 5). In contrast, a number of low molecular weight bands showing specific laddering pattern were observed in DNA samples obtained from the hepatocytes treated with high glucose indicating apoptosis.

#### Chromatin Condensation

Hoechst 33258 was used to study chromatin condensation, an important hallmark of apoptosis. Cells exposed to high glucose stress showed intense and increased fluorescent intensity which corresponds to nuclear condensation. Cells pre and co treated with morin were significantly more resistant than untreated cells to high glucose-induced apoptosis and hence showed no change in nuclear morphology (Fig. 5b).

#### Cell cycle analysis with cellular DNA content

Cell cycle analysis with cellular DNA content was performed by flow cytometry. Apoptotic cells were estimated by calculating the number of sub-diploid cells in the cell cycle histogram. When cells were exposed to glucose, apoptotic cells increased markedly. The number of sub-diploid cells after glucose treatment was found to be 15.52%. Significant increase in M1 peak was observed by 4.85 fold (P<0.001). Cells pre and co-treated with morin decreased the apoptotic DNA content to 2.43 fold (P<0.01) and 4.84 fold (P<0.001) respectively. Post treatment did not cause much significant effect, however the apoptotic DNA content was reduced to 12.17% (Fig. 5c).

## Discussion

Chronic hyperglycaemia is one of the hallmarks of diabetes and is implicated in the development of diverse complications, such as retinopathy, nephropathy, neuropathy and many more [34]. But fewer studies have focussed on the deleterious effect of chronic hyperglycaemia on liver, though it is the main site of glucose regulation. High glucose exhibits detrimental effect by deregulating metabolism and homeostasis of various cellular processes [5], [6]. High glucose in the blood plays a major role in the onset of the various liver diseases and may culminate into hepatopathy if untreated. And hence liver may be one of the most potent targets of hyperglycaemia as it stores glucose in the form of glycogen and is mainly involved in glucose metabolism. We focussed our study on the ameliorative effects of morin on high glucose induced cytotoxicity in primary hepatocytes. In our pilot studies we established an optimum cytotoxic dose of glucose for hepatocytes on the basis of results obtained from cell viability assays, ROS generation and various antioxidant assays. In addition to it we demonstrated the absence of any detrimental effects of similar concentration of mannitol to the hepatocytes, ruling out the possible involvement of hyperosmolarity.

High glucose has been shown to increase ROS in many cell types in patients with diabetes due to combination of increased production of ROS along with decreased antioxidant function [3], [35]. Glyceraldehyde auto-oxidation, increased aldose reductase reactivity and decrease in NADPH, increased production of superoxide from mitochondria, are some of the possible sources of increased ROS production. There are important glucose metabolism pathways which lead to the generation of ROS through excess glucose metabolites [36], [37]. Hence oxidative stress is a major contributor to the development of diabetic complications related to progression in liver [38]. Present study showed decreased antioxidant enzymes activity and the level of natural antioxidant GSH, with increased ROS generation under high glucose stress. Morin, a flavonoid and a known antioxidant, was able to restore the expression, activities of antioxidant enzymes and levels of GSH. Decreased levels of antioxidants and decreased expression of Mn-SOD, Catalase, GPX and GSH level have been reported under hyperglycaemic state (36–38). Studies have shown that hepatocytes are sensitive to oxidant damage, which has been attributed to low expression of antioxidant enzymes [35]. Excessive ROS generation can also be a causative factor for alteration in antioxidant enzymes expression and activity. Present study shows the involvement of various antioxidant genes under high glucose stress. m-RNA level and activity of various antioxidant enzymes like CuSOD, MnSOD, GPx and GR was altered under high glucose stress which is in accordance with earlier reports suggesting modulated expression of antioxidant genes during high glucose induced oxidative stress [38].

Oxidative stress or generation of ROS has been closely linked to apoptosis in many cell types. Co-relation of changes in mitochondrial membrane potential (MMP) and ROS generation have been recent reported [6], [39], [40]. Mitochondrial dysfunction has been an important hallmark of programmed cell death (Apoptosis). Hyperglycemia increases ROS production and apoptosis through inactivation of mitochondrial enzymes [41], [42]. Mitochondria are easily affected by oxidants as they themselves are major source of free radicals and hence have limited ability to cope with oxidative stress [40]–[43]. High glucose induced changes lead to mitochondrial failure, encompassing generation of ROS. The role of mitochondria in the execution of apoptosis is crucial since this organelle is involved in the release of pro-apoptotic factors. Decrease in mitochondrial membrane potential has been reported by many researchers in different cell types [44], [45]. Our result of decreased mitochondrial membrane potential in high glucose treated hepatocytes is in accordance with the earlier reports of mitochondrial dysfunction as a result of high glucose in different cell types [44]–[47]. Loss of mitochondrial integrity causes mitochondrial pore transition which facilitates release of apoptogenic proteins like AIF, Endo-G, Cyt-c and collapse of ΨΔm resulting in subsequent cell death. Translocation of mitochondrial resident proteins like AIF and Endo-G are believed to be involved in nuclear condensation and DNA damage leading to cell death. Present study reports the translocation of both proteins from mitochondria to nucleus and this is parallel with earlier researches reporting the translocation from mitochondria to nucleus under hyperglycaemic condition. Morin, as an efficient antioxidant, was found to exert strong inhibitory effect on ROS generation and hence helped in maintenance of mitochondrial integrity by maintaining mitochondrial membrane potential. While preventing mitochondrial depolarization, morin caused decline in the release of apoptogenic factors from mitochondria and increased expression of anti-apoptogenic factors like Bcl-2 and MnSOD. Among three different exposure conditions, pre-treatment with morin was found to have maximal ameliorative effects. Hydroxyl group plays an important role in beneficial properties rendered by morin. Hydroxyl group at the C-3 and C-5, besides at C-4 helps to quench free radicals generated and is considered contributory for its antioxidant activity [48]. While hydroxyl group at C-2 is responsible for its anti-peroxidative property. In view of our results obtained, morin can be considered as anti-apoptotic agent which modulates mitochondrial membrane potential and maintains the levels of apoptotic and anti-apoptotic proteins. Due to hydroxyl group placed at carbon 5 and 7 it is known to exhibit estrogenic activity which makes it useful even in cancer prevention [49].

The nuclear morphological features induced by high glucose in primary hepatocytes completely reflected the classic apoptotic morphological features of chromatin condensation, chromatin marginalization to the nuclear envelope and nuclear fragmentation to form apoptotic bodies. This was further confirmed by apoptotic DNA content as evident from PI staining. High glucose treated hepatocytes exhibited remarkable increase peak of sub G1 population. On treating high glucose stressed hepatocytes with morin, apoptotic population, DNA fragmentation and chromatin condensation were significantly decreased. Hence, morin was found to be effective in ameliorating mitochondria mediated apoptosis caused by high glucose stress in primary hepatocytes.

## Conclusions

In summary, we provide evidence for the first time that morin can protect hepatocytes from hyperglycemia-induced apoptosis and dysfunction. Morin induces changes associated with better cell survival and function, including inhibition in ROS generation, translocation of apoptotic proteins, up-regulation of antioxidant genes and Bcl-2 gene expression. These results suggest that the naturally occurring flavonoid morin may have anti-diabetic potential by promoting survival and functional integrity and survival of hepatocytes.

## Supporting Information

Figure S1 **Structure of morin: A flavonoid with chemical name 3,5,7,2′,4′ pentahydroxyflavone.**
(TIF)

Figure S2 **Different Treatment regimens of Morin to high glucose stressed cells in vitro.**
(TIF)
