# Supplementary figures and images for: Protective Role of Morin, a Flavonoid, against High Glucose Induced Oxidative Stress Mediated Apoptosis in Primary Rat Hepatocytes
Source: PLoS One. 2012 Aug 10;7(8):e41663. doi: 10.1371/journal.pone.0041663 (PMC3416810; doi:10.1371/journal.pone.0041663)

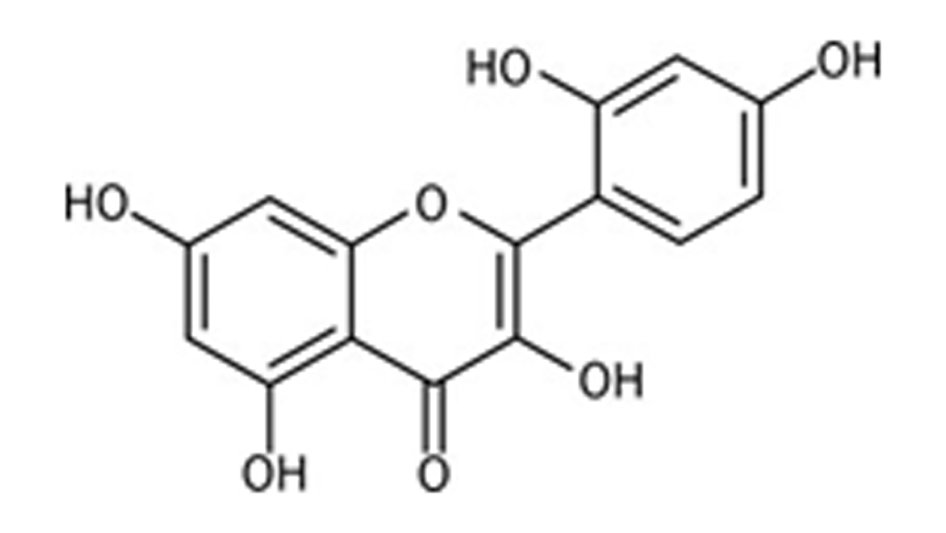

Supplement: Figure S1 — Structure of morin: A flavonoid with chemical name 3,5,7,2′,4′ pentahydroxyflavone. (TIF) [file pone.0041663.s001.tif]

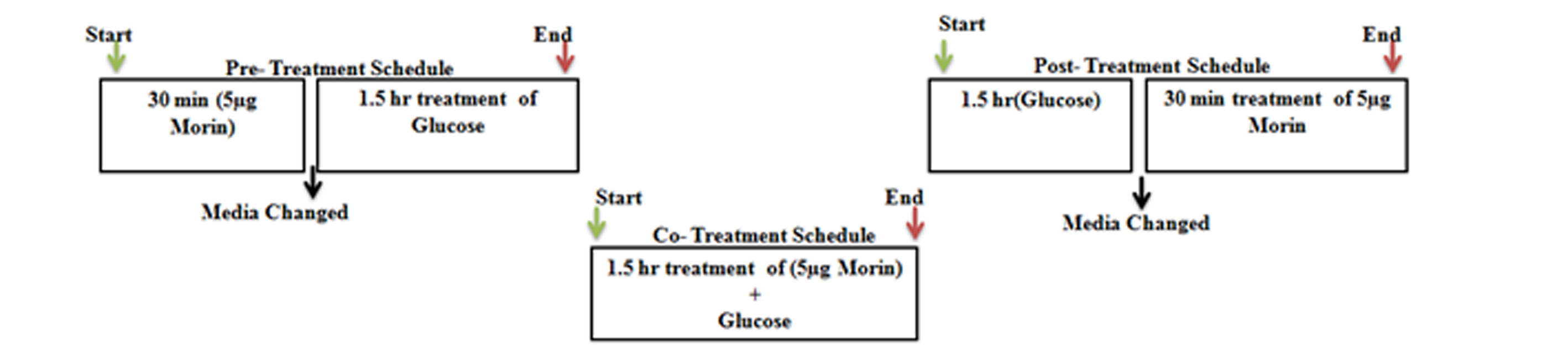

Supplement: Figure S2 — Different Treatment regimens of Morin to high glucose stressed cells in vitro. (TIF) [file pone.0041663.s002.tif]
